# Carbapenem-Resistant *Acinetobacter baumannii* Isolation and In-Hospital Mortality in Adults Hospitalized with COVID-19 in Romania: A Retrospective Single-Center Cohort Study

**DOI:** 10.3390/jcm15208093

**Published:** 2026-10-19

**Authors:** Cristiana Georgeta Bujor, Marilena Dinuti, Felicia Sfrijan

**Affiliations:** Department IV—Biochemistry and Pharmacology, Faculty of Medicine, “Victor Babeş” University of Medicine and Pharmacy Timișoara, Eftimie Murgu Square 2, 300041 Timișoara, Romania

**Keywords:** *Acinetobacter baumannii*, carbapenem resistance, COVID-19, colonization, antimicrobial stewardship, colistin, in-hospital mortality, retrospective cohort study

## Abstract

**Background/Objectives:** We assessed the frequency, timing and management of carbapenem-resistant *Acinetobacter baumannii* (CRAB) isolation and its association with in-hospital mortality in adults hospitalized with COVID-19. **Methods:** This retrospective single-center cohort study included all 395 adults admitted from 12 July to 25 September 2022. The primary analysis compared in-hospital mortality by CRAB isolation in the full cohort, with Firth-penalized adjustment for age and admission SpO_2_. Treatment and case-level data, complete for 95 chart-verified patients, were analyzed secondarily; isolates were classified by onset (NHSN criteria) and respiratory isolates adjudicated as infection or colonization. **Results:** CRAB was isolated in 33/395 patients (8.4%). Mortality was 36.4% (12/33) with CRAB isolation versus 11.6% (42/362) without (crude OR 4.35, 95% CI 1.81–10.02; adjusted OR 3.82, 95% CI 1.62–9.02). In the detailed-data subset (8/95 with CRAB), isolates were first detected on hospital days 2–6 and four were community-onset; four of seven respiratory isolates represented colonization. All eight received empirical carbapenem plus colistin after specimen collection, including full 9–10-day colistin-based courses in the four colonized patients. **Conclusions:** CRAB isolation was associated with higher in-hospital mortality after adjustment for baseline severity, but selective sampling precludes causal inference. Early detection suggests frequent carriage on admission; treatment of colonization was the main stewardship target, supporting admission screening and diagnostic stewardship.

## 1. Introduction

COVID-19 is a systemic infectious disease that can cause viral pneumonia, respiratory failure and extrapulmonary complications [1,2]. The present study covers the 2022 period in which Omicron BA.4/BA.5 lineages predominated [3]. Hospital-acquired bacterial organisms and antimicrobial exposure were important concerns throughout the pandemic, although culture sampling, case mix and timing substantially affect their reported frequency [4,5,6,7].

Bacterial co-infection at presentation is uncommon in COVID-19. A living meta-analysis estimated co-infection on admission at 3.5% and secondary bacterial infection later in the stay at 14.3%, while 72% of patients still received antibacterial drugs [4,5,6]. That mismatch—low documented bacterial infection, high empirical antibiotic use—created the ecological conditions in which multidrug-resistant Gram-negative bacilli could expand [7,8,9,10].

Carbapenem-resistant *Acinetobacter baumannii* is a priority antimicrobial-resistant pathogen [11,12,13]. Environmental persistence, device-associated colonization and several mechanisms of carbapenem resistance are described in the literature [14,15,16,17,18].

During the pandemic, CRAB caused outbreaks and marked increases in colonization and infection among patients with COVID-19, mostly in intensive care units (ICUs) [19,20,21,22,23,24,25], and a meta-analysis found that 96% of *Acinetobacter* isolates from patients with COVID-19 and bacterial infection were carbapenem-resistant [26]. However, these data come predominantly from mechanically ventilated ICU patients in the early pandemic waves, the incidence of hospital-acquired bacterial infection varied between waves [27], and patients treated on ordinary infectious-disease wards during the Omicron period—where most COVID-19 patients in later waves were actually treated—have been studied less. In Romania, published COVID-19 data on *A. baumannii* come mainly from ICUs [28,29]. Moreover, the clinical meaning of CRAB isolation depends on whether it reflects carriage present on admission or acquisition during the stay, as well as colonization or infection—distinctions that matter both for interpreting associations with outcomes and for antimicrobial stewardship.

We therefore examined CRAB isolation in all adults hospitalized with COVID-19 in a Romanian infectious diseases hospital during the Omicron BA.4/BA.5 period, assessing its association with in-hospital mortality in the full cohort and, in a chart-verified subset with detailed data, the timing and onset of isolation, the distinction between infection and colonization, and the antimicrobial treatment it triggered. Isolation denotes a laboratory finding made at any time during admission, not a baseline exposure, proven pneumonia or attributable cause of death.

## 2. Materials and Methods

### 2.1. Study Design and Setting

This retrospective, single-center cohort study was conducted in a tertiary infectious diseases hospital in Romania and included all 395 adults hospitalized with COVID-19 between 12 July and 25 September 2022, the local period of BA.5 circulation. Individual viral sequencing was not performed, so the period does not establish patient-level lineage. Event timing was recorded as hospital days counted from admission (day 1). Reporting follows STROBE (Supplementary Checklist S1) [30].

### 2.2. Ethics

The protocol was reviewed and approved by the Ethics Committee of the Clinical Hospital of Infectious Diseases and Pneumophysiology “Dr. Victor Babeș” Timișoara, Romania (approval no. 4711/20.05.2026); approval was granted retrospectively and specifically covers the secondary analysis of existing clinical records and publication of anonymized data for the period studied. The study was conducted in accordance with the Declaration of Helsinki (2013 revision) [31]. Because the analysis used only pre-existing clinical records, reported exclusively aggregated or irreversibly de-identified data and involved no additional procedures or patient contact, the committee waived the requirement for individual informed consent. Direct identifiers were removed before analysis.

### 2.3. Participants

The study cohort comprised all 395 hospitalized adults with SARS-CoV-2 infection confirmed by reverse-transcription polymerase chain reaction (RT-PCR). For all 395 patients, CRAB status, in-hospital mortality and core variables (age, sex, admission SpO_2_, ICU admission, microbiological sampling and major comorbidities) were extracted and source-verified; these patients constitute the primary analysis population. Detailed article-specific variables were collected and verified for 95 patients; for the remaining 300 patients, these non-core variables were not extracted, resulting in 100% missingness for those fields outside the detailed-data subset (Supplementary Table S7). These 95 patients constitute the detailed-data subset used for secondary analyses (Supplementary Figure S1). There was no matching.

### 2.4. Exposure, Microbiology and Definitions

The exposure was any recorded *Acinetobacter baumannii* isolate from a clinical specimen collected during the index admission. Species identification used MALDI-TOF MS. Meropenem and imipenem susceptibility was determined by broth microdilution and interpreted according to the EUCAST breakpoint tables applicable in 2022 (v12.0) [32]; isolates categorized as resistant to both agents were classified as CRAB. Individual carbapenem MIC values were not archived, and no additional confirmatory method for carbapenem resistance was documented. Colistin susceptibility was determined by broth microdilution. EUCAST v12.0 provides no *Acinetobacter* breakpoints for ampicillin-sulbactam or tigecycline; ampicillin-sulbactam inhibition zones and tigecycline MICs were recorded from laboratory reports and interpreted by the local laboratory using CLSI and FDA criteria, respectively (Supplementary Table S4). Because complete susceptibility panels were not archived, isolates were not categorized as multidrug- or extensively drug-resistant [17]. Cefiderocol was not tested.

The onset of each CRAB isolate was classified according to the National Healthcare Safety Network (NHSN) laboratory-identified event criteria as community-onset when the first positive specimen was collected on hospital day 1, 2 or 3 and as hospital-onset when it was collected on or after hospital day 4 [33]. Admission screening for CRAB carriage was not performed. Previous hospitalization, transfer from another healthcare facility, residence in a long-term care facility, antimicrobial exposure before admission and previous CRAB colonization or infection were not systematically recorded in the clinical records available for this study.

Microbiological sampling was clinician-directed: blood cultures and respiratory specimens (sputum or tracheal aspirates) were obtained at the discretion of the treating physicians, and no protocol-based surveillance cultures were performed. Respiratory CRAB isolates were adjudicated retrospectively from chart data according to IDSA/ATS definitions [34], considering new radiological infiltrates, compatible clinical signs, respiratory deterioration and semiquantitative culture growth, and were classified as hospital-acquired or ventilator-associated pneumonia or as respiratory colonization (Supplementary Table S5). Systemic infection and sepsis parameters were evaluated using standard clinical definitions and Sepsis-3 criteria [35].

### 2.5. Variables

For all 395 patients, the available variables were age, sex, admission SpO_2_, major comorbidities (cardiovascular disease, diabetes and chronic kidney disease), blood and respiratory culture sampling, CRAB status, ICU admission and vital status at discharge. In the detailed-data subset, additional variables comprised residence, body mass index, vaccination history, smoking, supplemental oxygen requirements, remdesivir [36], anakinra [37], systemic corticosteroids [38], named antibacterial courses, laboratory parameters, the hospital day of first positive specimen collection and total length of hospitalization. The primary outcome was all-cause in-hospital death.

For each CRAB case, the antimicrobial regimen, its start day relative to specimen collection and its total duration were extracted from patient charts (Supplementary Table S1). Empirical treatment of suspected severe hospital-acquired or healthcare-associated infection followed the institutional protocol, which, because of the high local prevalence of CRAB (supported by high regional and national carbapenem resistance rates in invasive *A. baumannii* isolates [29,39]), recommended a carbapenem plus colistin pending susceptibility results, followed by susceptibility-guided adjustment and a total duration of 7–10 days, in line with recommendations that empirical regimens reflect the local distribution of pathogens and their susceptibility [34].

### 2.6. Bias and Study Size

The study size was fixed by the number of admissions during the study period (N = 395) and the number of CRAB isolations; no a priori power calculation was performed. Including the full cohort in the primary analysis avoids the selection inherent in restricting the analysis to patients with complete article-specific data. The probability of culture sampling may depend on severity and outcome, so unsampled patients may have had undetected CRAB carriage. CRAB was recorded during hospitalization, while ICU care, treatment and survival accumulate over time; comparing ever-detected with never-detected patients is therefore subject to reverse causation, time-dependent confounding and immortal-time bias. These biases were examined by adjustment for baseline severity, by a day-6 landmark analysis and by sensitivity analyses (Section 2.7). No estimate is interpreted as a causal effect.

### 2.7. Statistical Analysis

Descriptive statistics are presented as counts and percentages for categorical variables and as medians with interquartile ranges (IQR) for continuous variables. Observed proportions are reported with 95% Wilson score confidence intervals. Categorical variables were compared with Fisher’s exact test, and continuous variables with the Mann–Whitney U test. Because the similarity of distribution shapes between groups was not formally verified, Mann–Whitney results are interpreted as evidence of a distributional difference (stochastic dominance) rather than of a difference in medians.

The primary analysis compared in-hospital mortality between patients with and without CRAB isolation in the full cohort (*N* = 395). The sample cross-product odds ratio is reported with the two-sided Fisher exact *p*-value and an exact conditional 95% confidence interval calculated using SciPy.stats.contingency.odds_ratio(kind = “conditional”). A Firth-penalized logistic regression model adjusted for two baseline severity markers measured before CRAB detection (age and admission SpO_2_) was used to estimate the adjusted odds ratio (aOR).

The following secondary and sensitivity analyses were performed: (1) the same crude and Firth-penalized analyses restricted to the detailed-data subset (n = 95), which constituted the primary analysis of the original submission; (2) a conditional day-6 landmark analysis among subset patients alive and hospitalized on day 6, with exposure defined as CRAB detection on or before day 6, to address immortal-time bias; a Cox proportional hazards model estimated the HR and 95% CI, with survival time measured from day 6 to in-hospital death or discharge; (3) multiple imputation by chained equations (MICE, m = 20 imputations) across the full cohort under a missing-at-random (MAR) assumption, imputing the article-specific covariates (such as inflammatory markers and detailed treatment timings) that were incomplete outside the subset, whereas CRAB status, mortality and core variables were complete and were not imputed; MICE used tree-based models in Miceforest, with 10 iterations. Predictors included CRAB status, mortality, age, sex, admission SpO_2_, ICU admission and major comorbidities, together with the article-specific laboratory, clinical and treatment variables; the mortality model fitted in each completed dataset included CRAB isolation and the imputed covariates, and estimates were pooled using Rubin’s rules; and (4) a comparison of patients with and without complete detailed data, including CRAB frequency (Supplementary Table S2). CRAB isolates in the subset were described by onset and adjudication category without formal testing. The false discovery rate (FDR) was controlled with the Benjamini–Hochberg (BH) procedure within five families: full-cohort baseline contrasts (Table 1; 9 tests), subset baseline contrasts (Table 2; 15 tests), treatment and complication contrasts (Table 3; 15 tests), secondary mortality contrasts (Table 4; 7 tests) and the primary and sensitivity estimates (Supplementary Table S6; 8 estimates). The full-cohort primary analysis was adopted during peer review, and the penalized, landmark, imputation and multiplicity analyses were added post hoc. All analyses other than the primary comparison are exploratory.

All statistical computations were executed using Python (version 3.10) with the SciPy, Statsmodels, and Miceforest libraries.

## 3. Results

### 3.1. Cohort Description

The full cohort comprised 395 patients (Supplementary Figure S1). The median age was 71 years (IQR 62–81), 269 (68.1%) were men, 35 (8.9%) were admitted to the ICU and 54 (13.7%) died in the hospital (Table 1). Detailed article-specific data were complete for 95 patients (detailed-data subset); missingness among the other 300 patients is summarized by variable in Supplementary Table S7. Patients with and without complete detailed data were similar in age, sex, admission SpO_2_, ICU admission, in-hospital mortality, culture sampling and major comorbidities (Supplementary Table S2). In the subset, the median age was 72 years (IQR 64–83), 68 (71.6%) were men, 46 (48.4%) had documented vaccination and 23 (24.2%) had obesity (Table 2).

### 3.2. Microbiology of A. baumannii

**Figure 1:**
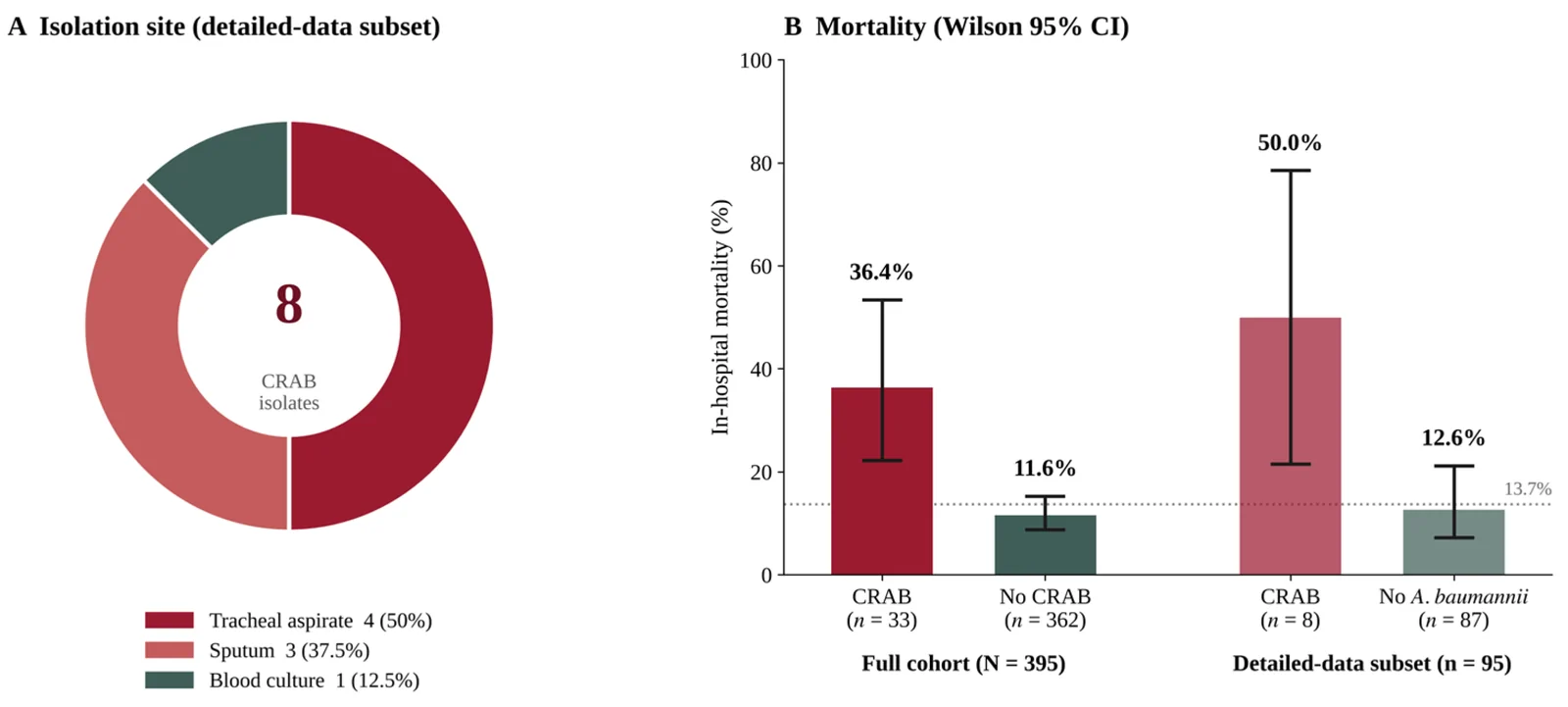
(**A**) Anatomical site of the eight CRAB isolates in the detailed-data subset (*n* = 95). (**B**) Crude in-hospital mortality with Wilson 95% confidence intervals in the full cohort (N = 395; primary analysis) and in the detailed-data subset. The dotted line marks the full-cohort mortality of 13.7%.

In the full cohort, CRAB was isolated in 33 of 395 patients (8.4%; Wilson 95% CI 6.0–11.6%): 8 in the detailed-data subset and 25 among the 300 patients without complete detailed data. No carbapenem-susceptible *A. baumannii* isolates were recorded. In the subset, eight of 95 patients (8.4%; Wilson 95% CI 4.3–15.7%) had a recorded CRAB isolate: four from tracheal aspirate, three from sputum and one from blood (Figure 1A). The first positive specimen was collected on hospital day 2 in three cases, day 3 in one, day 4 in one, day 5 in two and day 6 in one (median 3.5). By NHSN criteria, four isolates were community-onset (C3, C6, C7 and C8; days 2–3) and four were hospital-onset (C1, C2, C4 and C5; days 4–6). On retrospective adjudication, three respiratory isolates fulfilled the criteria for hospital-acquired or ventilator-associated pneumonia (C2, C4 and C6) and four were classified as colonization (C1, C3, C7 and C8). Three of the four colonization isolates were community-onset, whereas two of the three pneumonia isolates and the bloodstream isolate were hospital-onset; two of the four patients in each onset category died (Table 5; Supplementary Table S5).

Respiratory specimens were obtained from 28 of 95 subset patients (29.5%), and CRAB was isolated in seven (25.0%; sputum 3/16, tracheal aspirate 4/12; Supplementary Table S3). All eight isolates were resistant to meropenem and imipenem and susceptible to colistin (MIC ≤ 2 mg/L); ampicillin-sulbactam inhibition zones ranged from 16 to 21 mm and tigecycline MICs from 1.0 to 2.0 mg/L (Supplementary Table S4).

In the subset, a blood culture result was recorded for all 95 patients: 70 (73.7%) had no growth, and 25 had at least one reported organism, namely *Streptococcus pneumoniae* (7), *Escherichia coli* (4), *Enterococcus faecalis* (4), *Staphylococcus aureus* (3), *Klebsiella pneumoniae* (3), *Pseudomonas aeruginosa* (3) and *A. baumannii* (1). One CRAB patient (C2) grew *S. pneumoniae* from blood and was subsequently diagnosed with advanced HIV infection and *Pneumocystis jirovecii* pneumonia; another (C4) grew *E. coli*.

### 3.3. Antimicrobial Treatment and Complications in the Detailed-Data Subset

In the subset, carbapenem use was recorded for 8/8 patients with CRAB and 7/87 without *A. baumannii* isolation (Table 3). In every CRAB case, however, the carbapenem was started empirically after the first positive specimen had been collected, and no carbapenem administration before specimen collection was recorded during the index admission; this contrast therefore reflects treatment triggered by suspected infection, not exposure preceding CRAB detection. Anakinra (5/8 vs. 9/87; BH *q* = 0.007) and vancomycin (4/8 vs. 3/87; *q* = 0.005) were also more frequent among CRAB-positive patients.

All eight patients with CRAB isolation received empirical carbapenem plus colistin, started on the day the first positive specimen was collected. After the susceptibility report, the carbapenem was replaced by ampicillin-sulbactam (*n* = 6) or tigecycline (*n* = 2), and colistin was continued for a total of 10 days (9 days in C8) (Table 5; Supplementary Table S1). Cefiderocol was not administered. The same full course was given to the four patients whose isolates were adjudicated as colonization (C1, C3, C7 and C8), accounting for 39 of the 79 colistin treatment days administered to CRAB-positive patients; one of them (C8) had *C. difficile* infection recorded. Collection timestamps and laboratory notification times were not archived, precluding hours-level time-to-detection analyses.

*C. difficile* infection was recorded for 3/8 versus 5/87 patients; its onset date was not recorded (Table 3).

### 3.4. Outcomes

In the full cohort, in-hospital death occurred in 12 of 33 patients (36.4%) with CRAB isolation and in 42 of 362 (11.6%) without (crude OR 4.35, exact conditional 95% CI 1.81–10.02; Fisher exact *p* = 0.0005; Table 1; Figure 1B). After Firth-penalized adjustment for age and admission SpO_2_, the aOR was 3.82 (95% CI 1.62–9.02; *p* = 0.002). Median admission SpO_2_ was 88% in patients with CRAB isolation versus 93% in those without (*p* < 0.001).

Secondary analyses in the detailed-data subset gave consistent but imprecise estimates (Supplementary Table S6). Death occurred in 4/8 (50.0%) patients with CRAB isolation versus 11/87 (12.6%) without (crude OR 6.91, exact conditional 95% CI 1.08–41.79; *p* = 0.0199; Table 4); the Firth-adjusted OR was 5.12 (95% CI 0.88–29.84; *p* = 0.0688), and in the day-6 landmark analysis (n = 88; 4 deaths among 8 CRAB cases vs. 8 deaths among 80 controls), the hazard ratio was 5.67 (95% CI 1.63–19.72; *p* = 0.0063). Multiple imputation of article-specific covariates across the full cohort yielded a pooled OR of 5.82 (95% CI 0.94–36.02; *p* = 0.0584). Admission SpO_2_ was lower in CRAB-positive subset patients (87.5% vs. 92.0%; *p* = 0.016), and median length of hospitalization was 25.5 days (IQR 14.0–28.5) versus 8.0 days (IQR 5.0–14.0) (Hodges–Lehmann shift +11.5 days, 95% CI 3.2–19.8; *p* = 0.0124). After BH correction across the eight primary and sensitivity estimates, q-values were 0.0039 for the primary full-cohort crude analysis, 0.0084 for the full-cohort Firth model, 0.0168 for the day-6 landmark analysis, 0.0248 for length of stay, 0.0318 for the subset crude analysis, 0.0779 for MICE, 0.0786 for the subset Firth model and 0.2081 for the respiratory-sampled subset (Supplementary Table S6).

Univariable contrasts for ICU admission, cardiovascular disease, COVID-19 vaccination and concomitant therapies in the subset are shown in Table 4. Cardiorespiratory arrest was recorded as the immediate terminal mechanism for all fatal cases.

### 3.5. Individual CRAB Cases

Case-level timing, onset classification, treatment and adjudication for the eight CRAB cases are summarized in Table 5 and detailed in Supplementary Tables S1 and S5.

## 4. Discussion

### 4.1. Principal Findings

In this cohort of 395 adults hospitalized with COVID-19, predominantly in infectious-disease wards during the Omicron BA.4/BA.5 period, CRAB was isolated in 33 patients (8.4%). Crude in-hospital mortality was higher among patients with CRAB isolation, and the association was retained after adjustment for age and admission SpO_2_; estimates in the detailed-data subset were consistent but imprecise. Three further findings qualify this association. First, half of the isolates in the subset were community-onset, suggesting that CRAB carriage was often present on admission rather than acquired during the current stay. Second, four of the seven respiratory isolates represented colonization, yet all colonized patients received a full course of colistin-based therapy. Third, carbapenem therapy followed rather than preceded CRAB detection in every case. Selective culture sampling, pre-detection severity differences and day-level timing prevent attributing death, prolonged stay or antimicrobial use to CRAB.

### 4.2. Comparison with Previous Work

CRAB has been one of the most consistently reported resistant pathogens in COVID-19. In a meta-analysis of 148 studies, 96% of *Acinetobacter* isolates from patients with COVID-19 and bacterial infection were carbapenem-resistant, and ICU care was associated with higher odds of resistance [26]. During the early waves, outbreaks and sharp increases in CRAB colonization and infection were reported, predominantly from ICUs, in Italy, Switzerland, Israel and the United States [21,22,23,24]. In a multicenter Italian ICU study, CRAB colonization rose from 5.1 to 36.8 and infection from 5.1 to 26.4 per 10,000 patient-days compared with the pre-pandemic period [22], and in a Rome ICU the cumulative incidence of *A. baumannii* acquisition was 36.8%, with prior carbapenem use as the only independent risk factor [25]. Other ICU series reported a high CRAB burden and high mortality among mechanically ventilated patients [19,20,40]. In a systematic review, all reported CRAB outbreaks in COVID-19 involved intensive care settings, and inadequate use of personal protective equipment and carbapenem overuse were considered key drivers [24].

The burden of hospital-acquired infection also differed across pandemic waves. In 26 Israeli hospitals, the incidence of hospital-acquired sentinel bacteria among patients with COVID-19 was highest during the first wave and lower in later waves despite a higher proportion of severely ill patients, suggesting that healthcare practices rather than patient characteristics drove most infections [27]. In Romania, published data come largely from ICUs during the pre-Omicron waves: in a COVID-19 ICU in Târgu Mureș (August 2020–January 2021), *A. baumannii* accounted for 31.1% of isolates from secondary infections, mostly multidrug-resistant, and overall mortality was 84.4% [28], and a systematic review of Romanian studies found that about three-quarters of reported *Acinetobacter* isolates were multidrug-resistant [29], against a national background of high carbapenem resistance in invasive *Acinetobacter* isolates [39]. Our cohort differs from these settings: patients were treated predominantly in infectious-disease wards (ICU admission in 8.9% of the full cohort), during the Omicron BA.4/BA.5 period, and with clinician-directed rather than surveillance cultures, so CRAB frequency cannot be compared with ICU incidence or surveillance-based estimates.

Against this background, the present study adds a full-cohort estimate of the mortality associated with CRAB isolation outside the ICU in the Omicron period, case-level timing showing that half of the isolates were community-onset, and adjudication showing that most respiratory isolates represented colonization that was nevertheless treated. The higher crude mortality among patients with CRAB isolation should be interpreted in light of their lower admission SpO_2_, the co-pathogens and comorbid conditions recorded in several cases and the predominance of colonization among respiratory isolates; no estimate of attributable CRAB mortality can be obtained. Global surveillance continues to recognize carbapenem-resistant *Acinetobacter* as a critical-priority pathogen [12,13,39,41].

### 4.3. Timing and Origin of CRAB Isolation

CRAB was first isolated early in hospitalization (days 2–6), and half of the isolates met NHSN criteria for community-onset [33]. In the absence of admission screening, this pattern suggests that some patients were already colonized on admission, which is plausible in a country where carbapenem resistance in *Acinetobacter* is highly prevalent [29,39]. Because previous healthcare exposure and CRAB colonization history were not recorded (Section 2.4), we could not distinguish carriage imported at admission from acquisition during the current stay. Conversely, a hospital-onset classification does not prove nosocomial acquisition, because carriage present at admission may be detected only once sampling begins. The origin of CRAB therefore cannot be reliably established; admission screening of high-risk patients, linked to records of previous healthcare exposure, would be needed to do so.

All eight patients received empirical colistin in addition to a carbapenem before susceptibility results were available. This regimen reflected the institutional protocol for suspected severe hospital-acquired or healthcare-associated infection, which was designed for a setting with a high local prevalence of CRAB, in line with recommendations that empirical regimens be based on local resistance data [34]. The protocol was applied even when the first positive specimen was collected on hospital day 2 or 3, that is, to isolates classified as community-onset. Nevertheless, the systematic addition of colistin may also indicate that clinicians suspected multidrug-resistant infection on clinical grounds, which is consistent with the possibility of pre-existing colonization; because the reasons for each prescription were not documented beyond the protocol, this cannot be verified.

### 4.4. Antimicrobial Stewardship and Treatment of Colonization

Antibacterials were recorded for 67/95 patients (70.5%) in the subset, a proportion comparable to that reported in a meta-analysis of antibiotic prescribing in COVID-19 [42] and much higher than the frequency of documented bacterial infection [4]; prescribing should be guided by the indication, confirmed infection status, current susceptibility results and relevant guidance [43,44]. The association between carbapenem use and CRAB isolation should not be interpreted as a stewardship finding: in every case, the carbapenem was started after the CRAB-positive specimen had been collected, as empirical therapy for suspected infection, and it was discontinued once resistance was reported, consistent with trial evidence that adding meropenem to colistin does not improve outcomes [45]; colistin-based combinations remained the backbone of therapy [46,47]. Antibiotic exposure before admission was not captured, so a contribution of earlier carbapenem exposure, a recognized risk factor for *A. baumannii* acquisition [25], cannot be excluded.

The most relevant stewardship finding is that all four patients whose respiratory isolates were adjudicated as colonization received the full 9–10-day course of colistin, combined first with a carbapenem and then with ampicillin-sulbactam or tigecycline, accounting for nearly half (39 of 79) of the colistin treatment days in CRAB-positive patients. None of these four patients had a new radiological infiltrate or respiratory deterioration (Supplementary Table S5), no indication beyond the institutional protocol was documented, and colistin was continued for the full course after the susceptibility report rather than stopped once infection was not confirmed. Respiratory colonization does not require antimicrobial treatment, which should be reserved for patients with clinical, radiological and microbiological evidence of infection [34,44]. Unnecessary colistin exposure carries risks of nephrotoxicity and neurotoxicity [46], selects for further resistance and disrupts the intestinal microbiota; *C. difficile* infection was recorded in one of the four colonized patients, although its timing relative to treatment could not be determined [48]. Treatment decisions were made at the bedside before the retrospective adjudication, when distinguishing colonization from infection is difficult because the infiltrates and respiratory deterioration of COVID-19 pneumonia overlap with those of bacterial pneumonia. These observations point to opportunities for diagnostic stewardship—obtaining respiratory cultures only when bacterial pneumonia is clinically suspected and interpreting semiquantitative growth together with clinical and radiological findings—and for a structured reassessment of antimicrobial therapy at 48–72 h, with discontinuation when infection is not confirmed.

### 4.5. Other Exploratory Contrasts

Anakinra was recorded in 5/8 CRAB-positive patients and 9/87 others, and *C. difficile* infection in 3/8 and 5/87. The indications and timing of these events relative to CRAB detection and antimicrobial exposure were not available; severity-related confounding is plausible, and neither contrast can establish a drug–pathogen or antibiotic–infection relationship [37,48].

In the subset, three of 46 vaccinated patients and 12 of 49 unvaccinated patients died. This unadjusted secondary contrast may be influenced by age, comorbidities, prior infection, health-care access and illness severity; it is not an estimate of vaccine effectiveness.

### 4.6. Limitations

The primary analysis included the full cohort, but detailed treatment, laboratory and timing data were complete for only 95 patients, so the secondary analyses remain susceptible to selection, even though patients with and without detailed data were similar in the characteristics compared (Supplementary Table S2). Microbiological sampling was clinician-directed, and no admission or surveillance screening was performed: undetected CRAB carriage among unsampled patients is likely, CRAB frequency is not a prevalence estimate, and the origin of isolates (imported or hospital-acquired) cannot be established. Previous healthcare exposure and CRAB colonization history were not recorded. Detection was assessed at any time during admission; despite adjustment and the landmark analysis, ever-detected comparisons remain vulnerable to reverse causality and selective sampling, and ICU entry dates were not recorded in the source charts, preventing the modeling of ICU admission as a time-varying covariate. Only two covariates were included in the penalized models, and residual confounding by severity is likely. The MICE estimate relies on covariates that were not extracted for 300 patients and therefore depends strongly on the missing-at-random assumption. The sensitivity analyses were post hoc (Section 2.7).

Respiratory isolates were adjudicated retrospectively from chart data rather than prospectively [34], and misclassification between colonization and infection is possible. The odds ratio for mortality is crude, imprecise and cannot be used for individual clinical risk prediction.

Individual carbapenem MICs were not archived, no independent confirmatory method for carbapenem resistance was documented, and EUCAST v12.0 provides no *Acinetobacter* breakpoints for ampicillin-sulbactam or tigecycline. Molecular carbapenemase data are absent, so OXA-type mechanisms could not be confirmed. Event timing was available only at hospital-day resolution, and *C. difficile* onset dates were not recorded. Exploratory comparisons involve many variables; Benjamini–Hochberg q-values are reported, and no exploratory contrast should be read as confirmatory. The BA.5 designation refers to a surveillance period, not sequenced patient isolates.

### 4.7. Implications

If confirmed in larger cohorts with admission screening, systematic culture surveillance and time-stamped exposure records, these observations support screening high-risk patients for CRAB carriage on admission, to distinguish imported from hospital-acquired CRAB and guide infection prevention; diagnostic stewardship and structured reassessment of therapy to avoid treating respiratory colonization; and prompt carbapenem de-escalation once susceptibility results are available. The present data do not show that surveillance or stewardship interventions change clinical outcomes.

## 5. Conclusions

In a cohort of 395 adults hospitalized with COVID-19, CRAB was isolated in 33 patients (8.4%), and crude in-hospital mortality was higher among those with CRAB isolation; after adjustment for baseline severity, the association was retained. In the detailed-data subset, half of the isolates were community-onset and most respiratory isolates represented colonization that was nonetheless treated with colistin-based regimens. Incomplete microbiological ascertainment and differences in initial severity preclude causal conclusions. Admission screening, diagnostic stewardship to avoid treating colonization and larger studies with systematic surveillance are warranted.

## Figures and Tables

**Table 1:** Baseline characteristics, microbiological sampling and outcomes of the full cohort (*N* = 395), according to CRAB isolation.

Variable	Full Cohort (*N* = 395)	CRAB (*n* = 33)	No CRAB (*n* = 362)	Descriptive
Age, years	71 (62–81)	71 (63–82)	71 (62–81)	*p* = 0.812
Male sex	269 (68.1)	22 (66.7)	247 (68.2)	*p* = 0.847
Admission SpO_2_, %	93 (90–96)	88 (85–91)	93 (91–96)	*p* < 0.001
Cardiovascular disease	211 (53.4)	18 (54.5)	193 (53.3)	*p* = 1.000
Diabetes, any type	190 (48.1)	18 (54.5)	172 (47.5)	*p* = 0.471
Chronic kidney disease	65 (16.5)	6 (18.2)	59 (16.3)	*p* = 0.806
Blood culture obtained	395 (100)	33 (100)	362 (100)	—
Respiratory specimen obtained	106 (26.8)	25 (75.8)	81 (22.4)	*p* < 0.001
ICU admission	35 (8.9)	8 (24.2)	27 (7.5)	*p* = 0.005
In-hospital death	54 (13.7)	12 (36.4)	42 (11.6)	*p* < 0.001

Values are *n* (%) or median (IQR). Unadjusted *p*-values from Fisher’s exact test (categorical variables) or the Mann–Whitney U test (continuous variables). Benjamini–Hochberg correction was applied across the nine tested contrasts (blood culture was obtained in all patients and was not tested). For admission SpO_2_, *q* < 0.003; for respiratory sampling, *q* < 0.001; for mortality, *q* ≤ 0.0022; and for ICU admission, *q* = 0.0105. CRAB, carbapenem-resistant *A. baumannii*; ICU, intensive care unit; SpO_2_, peripheral oxygen saturation.

**Table 2:** Baseline characteristics of the detailed-data subset (*n* = 95), according to *A. baumannii* isolation.

Variable	Subset (*n* = 95)	CRAB (*n* = 8)	No *A. baumannii* (*n* = 87)	Descriptive
Age, years	72 (64–83)	71 (63–85)	72 (64–82)	*p* = 0.963
Male sex	68 (71.6)	4 (50.0)	64 (73.6)	*p* = 0.222
Urban residence	54 (56.8)	4 (50.0)	50 (57.5)	*p* = 0.722
BMI, kg/m^2^	26.7 (24.5–29.6)	28.9 (24.9–31.6)	26.7 (24.4–29.5)	*p* = 0.406
Obesity (BMI ≥ 30)	23 (24.2)	3 (37.5)	20 (23.0)	*p* = 0.396
Any COVID-19 vaccine	46 (48.4)	4 (50.0)	42 (48.3)	*p* = 1.000
Current smoking	21 (22.1)	1 (12.5)	20 (23.0)	*p* = 0.680
Hypertension	70 (73.7)	6 (75.0)	64 (73.6)	*p* = 1.000
Cardiovascular disease	53 (55.8)	4 (50.0)	49 (56.3)	*p* = 0.730
Diabetes, any type	48 (50.5)	5 (62.5)	43 (49.4)	*p* = 0.715
COPD	12 (12.6)	1 (12.5)	11 (12.6)	*p* = 1.000
Chronic kidney disease	17 (17.9)	1 (12.5)	16 (18.4)	*p* = 1.000
Chronic liver disease	13 (13.7)	1 (12.5)	12 (13.8)	*p* = 1.000
Neurological disease	22 (23.2)	1 (12.5)	21 (24.1)	*p* = 0.678
Admission SpO_2_, %	92 (91–95)	87.5 (85.0–90.3)	92 (91–95)	*p* = 0.016

Values are *n* (%) or median (IQR). Unadjusted *p*-values from Fisher’s exact test (categorical variables) or the Mann–Whitney U test (continuous variables). After Benjamini–Hochberg correction across the 15 contrasts in this table, *q* = 0.24 for admission SpO_2_ and *q* = 1.000 for all other variables. BMI, body mass index; COPD, chronic obstructive pulmonary disease; CRAB, carbapenem-resistant *A. baumannii*.

**Table 3:** Treatments, selected complications and admission laboratory values in the detailed-data subset (*n* = 95).

Variable	CRAB (*n* = 8)	No *A. baumannii* (*n* = 87)	Descriptive
Supplemental oxygen	7 (87.5)	54 (62.1)	*p* = 0.252
Oxygen duration, days	12 (2–16)	2 (0–7)	*p* = 0.021
Systemic corticosteroid	6 (75.0)	64 (73.6)	*p* = 1.000
Remdesivir	4 (50.0)	46 (52.9)	*p* = 1.000
Anakinra	5 (62.5)	9 (10.3)	*p* = 0.002
Any antibacterial	8 (100)	59 (67.8)	*p* = 0.100
Carbapenem †	8 (100)	7 (8.0)	*p* < 0.001
Vancomycin	4 (50.0)	3 (3.4)	*p* = 0.001
ICU admission	2 (25.0)	7 (8.0)	*p* = 0.166
*C. difficile* infection	3 (37.5)	5 (5.7)	*p* = 0.018
Cardiorespiratory arrest	4 (50.0)	11 (12.6)	*p* = 0.020
Leukocytes, ×10^9^/L	12.9 (10.6–15.7)	9.0 (5.8–12.6)	*p* = 0.082
CRP, mg/L	91 (65–148)	51 (18–108)	*p* = 0.124
Procalcitonin, ng/mL	0.44 (0.11–0.80)	0.14 (0.07–0.80)	*p* = 0.288
Ferritin, ng/mL	769 (364–1125)	445 (270–971)	*p* = 0.215

Values are *n* (%) or median (IQR). Descriptive comparisons show unadjusted *p*-values computed using Fisher’s exact test for categorical variables and the Mann–Whitney U test for continuous variables. After Benjamini–Hochberg correction across the 15 contrasts in this table, *q* < 0.001 for carbapenem, *q* = 0.005 for vancomycin and *q* = 0.007 for anakinra; *q* = 0.053 for oxygen duration, *C. difficile* infection and cardiorespiratory arrest; and *q* > 0.15 for all other variables. All comparisons are unadjusted and descriptive; they do not establish independent risk or causal relationships. † In all CRAB cases, the carbapenem was started after collection of the first CRAB-positive specimen; this contrast does not represent exposure preceding detection.

**Table 4:** Univariable associations with in-hospital death in the detailed-data subset (*n* = 95).

Exposure	Deaths/Exposed	Deaths/Unexposed	Crude OR (Exact 95% CI)	Fisher Exact *p* (BH q)
CRAB isolation	4/8 (50.0%)	11/87 (12.6%)	6.91 (1.08–41.79)	0.020 (secondary)
ICU admission	4/9 (44.4%)	11/86 (12.8%)	5.45 (0.91–29.21)	0.032 (0.113)
Cardiovascular disease	12/53 (22.6%)	3/42 (7.1%)	3.80 (0.92–22.30)	0.049 (0.115)
Any COVID-19 vaccine	3/46 (6.5%)	12/49 (24.5%)	0.22 (0.04–0.89)	0.023 (0.113)
Male sex	12/68 (17.6%)	3/27 (11.1%)	1.71 (0.41–10.26)	0.544 (0.544)
Obesity	5/23 (21.7%)	10/72 (13.9%)	1.72 (0.41–6.41)	0.511 (0.544)
Diabetes	10/48 (20.8%)	5/47 (10.6%)	2.21 (0.62–8.94)	0.261 (0.365)
Any antibacterial	13/67 (19.4%)	2/28 (7.1%)	3.13 (0.63–30.30)	0.217 (0.365)

Odds ratios are sample cross-product estimates with exact conditional (Cornfield) 95% confidence intervals, which may not coincide exactly with the two-sided Fisher exact *p*-value. *q*-values are Benjamini–Hochberg-adjusted across the seven secondary contrasts. All estimates are unadjusted and exploratory and do not identify independent or causal predictors.

**Table 5:** De-identified case series of the eight patients with CRAB isolation. Treatment start refers to empirical carbapenem plus colistin, begun after collection of the first positive specimen; after susceptibility reporting, the carbapenem was replaced by ampicillin-sulbactam (tigecycline in C6 and C8), and colistin was continued throughout (10 days; 9 days in C8).

ID	Age/sex	Vaccine	Site	ICU	Anakinra	C. Diff.	LOS	Outcome	First CRAB Isolation (Hospital Day; Onset)	Empirical Carbapenem + Colistin Start (Hospital Day)	Respiratory Adjudication
**C1**	67 M	None	Tracheal asp.	No	No	No	26	Survived	Day 5 (HO)	Day 5	Colonization
**C2**	47 F	2-dose BNT162b2	Tracheal asp.	Yes	Yes	No	25	Survived *	Day 6 (HO)	Day 6	VAP (Pneumonia)
**C3**	75 F	None	Tracheal asp.	No	No	No	33	Survived †	Day 2 (CO)	Day 2	Colonization
**C4**	86 F	2-dose BNT162b2	Sputum	No	Yes	Yes	19	Died	Day 5 (HO)	Day 5	HAP (Pneumonia)
**C5**	85 M	None	Blood	Yes	Yes	Yes	30	Died	Day 4 (HO)	Day 4	Primary BSI
**C6**	64 M	3-dose BNT162b2	Sputum	No	Yes	No	14	Died	Day 3 (CO)	Day 3	HAP (Pneumonia)
**C7**	88 F	None	Tracheal asp.	No	Yes	No	28	Died	Day 2 (CO)	Day 2	Colonization
**C8**	59 M	3-dose BNT162b2	Sputum	No	No	Yes	10	Survived	Day 2 (CO)	Day 2	Colonization

* Newly diagnosed advanced HIV infection with *Pneumocystis jirovecii* pneumonia. † Concurrent pulmonary embolism. BSI, bloodstream infection; CO, community-onset (first positive specimen on hospital day 1–3); HAP, hospital-acquired pneumonia; HO, hospital-onset (hospital day ≥ 4), according to NHSN criteria [33]; ICU, intensive care unit; LOS, length of stay (days); VAP, ventilator-associated pneumonia.

## Data Availability

De-identified clinical and laboratory data supporting the findings are available from the corresponding author upon reasonable request, subject to institutional approval, data-sharing policies, and applicable privacy requirements.

## Supplementary Materials

The following supporting information can be downloaded at https://www.mdpi.com/article/10.3390/jcm15208093/s1: Figure S1: Flow diagram of the study cohort and the detailed-data subset; Table S1: First recorded CRAB isolation by hospital day, antimicrobial treatment sequence, and relative timing among the eight CRAB cases in the detailed-data subset; Table S2: Comparison of patients with and without complete detailed data (detailed-data subset vs. remainder of the full cohort), including CRAB status; Table S3: Microbiological sampling denominators in the detailed-data subset (*n* = 95); Table S4: Laboratory susceptibility methodology and phenotype; Table S5: Respiratory infection versus colonization assessment (retrospective chart-based adjudication according to IDSA/ATS definitions; C5, bloodstream isolate, not included); Table S6: Primary (full-cohort) and secondary or sensitivity analyses of the association between CRAB isolation and outcomes; Table S7: Variable-specific summary of missing data in the full cohort (*N* = 395); Checklist S1: STROBE statement—checklist of items for cohort studies. The aggregate categorical counts reported in Table 1, Table 2, Table 3, Table 4 and Table 5 permit verification of the corresponding 2 × 2 comparisons. Individual-level data are not included because their release is subject to institutional data-sharing and privacy requirements.

## Abbreviations

The following abbreviations are used in this manuscript:

aOR	adjusted odds ratio
ATS	American Thoracic Society
BH	Benjamini–Hochberg
BMI	body mass index
BSI	bloodstream infection
CI	confidence interval
CLSI	Clinical and Laboratory Standards Institute
CO	community-onset
COPD	chronic obstructive pulmonary disease
COVID-19	coronavirus disease 2019
CRAB	carbapenem-resistant *Acinetobacter baumannii*
CRP	C-reactive protein
EUCAST	European Committee on Antimicrobial Susceptibility Testing
FDA	U.S. Food and Drug Administration
FDR	false discovery rate
HAP	hospital-acquired pneumonia
HIV	human immunodeficiency virus
HO	hospital-onset
HR	hazard ratio
ICU	intensive care unit
IDSA	Infectious Diseases Society of America
IQR	interquartile range
LOS	length of stay
MALDI-TOF MS	matrix-assisted laser desorption/ionization time-of-flight mass spectrometry
MAR	missing at random
MIC	minimum inhibitory concentration
MICE	multiple imputation by chained equations
NHSN	National Healthcare Safety Network
OR	odds ratio
RT-PCR	reverse-transcription polymerase chain reaction
SARS-CoV-2	severe acute respiratory syndrome coronavirus 2
SpO_2_	peripheral oxygen saturation measured by pulse oximetry
STROBE	Strengthening the Reporting of Observational Studies in Epidemiology
VAP	ventilator-associated pneumonia
