# Supplementary material for: Carbapenem-Resistant Acinetobacter baumannii Isolation and In-Hospital Mortality in Adults Hospitalized with COVID-19 in Romania: A Retrospective Single-Center Cohort Study
Source: J Clin Med. 2026 Oct 19;15(20):8093. doi: 10.3390/jcm15208093 (PMC13705631; doi:10.3390/jcm15208093)
Supplement: Supplementary file 1 [file jcm-15-08093-s001.zip › jcm-4593430-supplementary.pdf]

Supplementary Materials

Supplementary Figure S1. Flow diagram of the study cohort and the detailed-data subset. BSI, bloodstream infection; CRAB, carbapenem-resistant *Acinetobacter baumannii*; MICE, multiple imputation by chained equations.

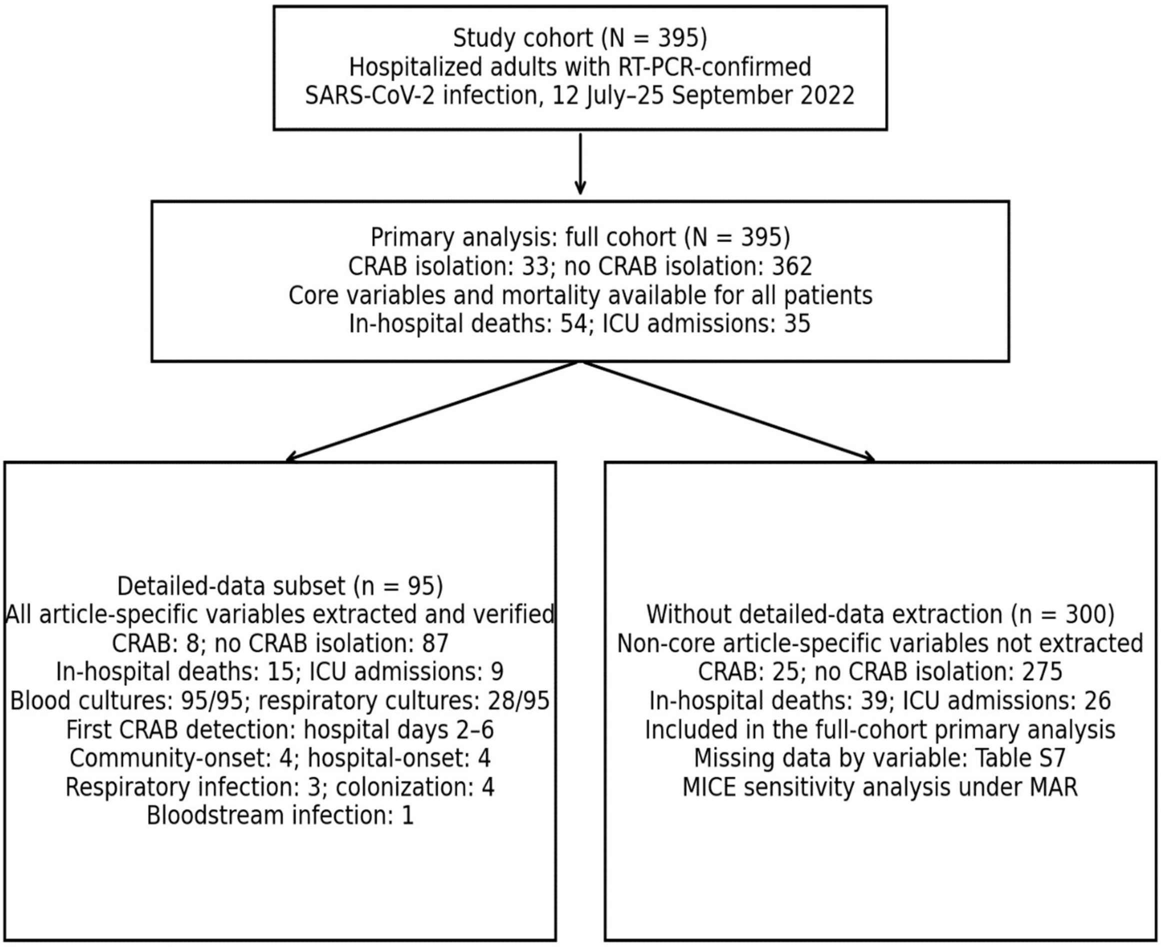

Supplementary Checklist S1: STROBE statement — checklist of items for cohort studies

Checklist for the revised manuscript. The checklist follows von Elm et al. (https://doi.org/10.1016/S0140-6736(07)61602-X).

STROBE item	Location in manuscript
Title and abstract — indicate the study's design with a commonly used term	Title; Abstract (Methods)
Abstract — informative and balanced summary of what was done and found	Structured abstract
Background — scientific background and rationale	§1 Introduction
Objectives — specific objectives, including any prespecified hypotheses	Last paragraph of §1

STROBE item	Location in manuscript
Study design — present key elements of study design early	§2.1
Setting — locations, dates, periods of recruitment and data collection	§2.1; admissions 12 July–25 September 2022; BA.5 circulation period, no patient-level sequencing
Participants — eligibility, sources and methods of selection, follow-up	§2.3 and §3.1; Supplementary Figure S1 (flow diagram); Table S2 (patients with vs. without complete detailed data); Table S7 (missing data by variable)
Variables — clearly define outcomes, exposures, predictors, confounders	§2.4–2.5
Data sources / measurement — sources of data and methods of assessment	§2.4–2.5; chart review and laboratory records; hospital day of first CRAB isolation, onset category (NHSN criteria) and treatment start for all eight subset cases (Table 5; Table S1)
Bias — describe any efforts to address potential sources of bias	§2.6 and §4.6; selection avoided in the primary analysis by including the full cohort; comparison of patients with and without complete detailed data (Table S2) and multiple imputation; non-uniform sampling (Table S3) and time-dependent bias (day-6 landmark) examined; residual bias discussed
Study size — explain how the study size was arrived at	§2.6; all 395 admissions during the study period (33 CRAB isolations); detailed-data subset of 95 patients (eight CRAB isolations); no a priori power calculation
Quantitative variables — explain how quantitative variables were handled	§2.7; medians and IQR; Mann–Whitney U results interpreted as distributional differences because similarity of distribution shapes was not verified
Statistical methods — all statistical methods, including those for confounding	§2.7; primary analysis in the full cohort: crude OR with exact conditional CI and Fisher exact test, and Firth penalized logistic regression adjusted for age and admission SpO_2; same analyses in the detailed-data subset (secondary); Benjamini–Hochberg FDR control
Subgroups / interactions / missing data / loss to follow-up / sensitivity	§2.3, §2.6 and §2.7; comparison of patients with and without complete detailed data (Table S2); missing data by variable (Table S7); MICE (m = 20) under MAR; day-6 landmark; respiratory-sampled subset (Table S6)

STROBE item	Location in manuscript
Participants — numbers at each stage (Figure S1 flow)	§3.1; Figure S1: 395 in the study cohort, all included in the primary analysis (33 CRAB, 362 no CRAB); 95 with complete detailed data (8 CRAB, 87 no *A. baumannii* isolation) and 300 without (25 CRAB)
Descriptive data — characteristics of study participants	Tables 1 and 2; §3.1
Outcome data — numbers of outcome events or summary measures	§3.4; Tables 1 and 4; Figure 1B (crude mortality proportions in the full cohort and the detailed-data subset)
Main results — unadjusted estimates and precision (95% CI)	§3.4; Table 1; full-cohort crude mortality OR 4.35 (exact conditional CI 1.81–10.02); Firth-adjusted OR 3.82 (1.62–9.02); subset estimates in Table 4 and Table S6; no attributable effect estimated
Other analyses — other analyses done (subgroups, interactions, sensitivity)	§3.2–3.5; Tables 3–5, Tables S1, S5 and S6; onset classification (NHSN); treatment followed specimen collection in all eight cases; treatment of colonization; post hoc sensitivity analyses
Key results — summarized with reference to study objectives	§4.1; §5
Limitations — limitations, potential bias, imprecision, multiplicity	§4.6; clinician-directed sampling without admission screening, unrecorded previous healthcare exposure, selection in subset analyses, hospital-day timing resolution, imprecision, post hoc sensitivity analyses and multiple comparisons
Interpretation — overall interpretation considering objectives, limitations, other studies	§4.2–4.7
Generalizability — external validity of the study results	§4.6–4.7; single-center cohort treated predominantly on infectious-disease wards in the Omicron period; CRAB frequency is not a prevalence estimate
Funding — source of funding and role of funders	Funding statement (back matter)

Supplementary Table S1. First recorded CRAB isolation by hospital day, antimicrobial treatment sequence, and relative timing among the eight CRAB cases in the detailed-data subset.

Case ID	Isolation site	First isolation day (from admission)	Empirical carbapenem → replacement after AST	Timing relative to first positive specimen	Start day of carbapenem + colistin / total duration
C1	Tracheal aspirate	Day 5	Meropenem → ampicillin-sulbactam	After specimen collection	Day 5 / 10 days

Case ID	Isolation site	First isolation day (from admission)	Empirical carbapenem → replacement after AST	Timing relative to first positive specimen	Start day of carbapenem + colistin / total duration
				(same hospital day)	
C2	Tracheal aspirate	Day 6	Ertapenem → ampicillin-sulbactam	After specimen collection (same hospital day)	Day 6 / 10 days
C3	Tracheal aspirate	Day 2	Meropenem → ampicillin-sulbactam	After specimen collection (same hospital day)	Day 2 / 10 days
C4	Sputum	Day 5	Meropenem → ampicillin-sulbactam	After specimen collection (same hospital day)	Day 5 / 10 days
C5	Blood	Day 4	Meropenem → ampicillin-sulbactam	After specimen collection (same hospital day)	Day 4 / 10 days
C6	Sputum	Day 3	Meropenem → tigecycline	After specimen collection (same hospital day)	Day 3 / 10 days
C7	Tracheal aspirate	Day 2	Meropenem → ampicillin-sulbactam	After specimen collection (same hospital day)	Day 2 / 10 days
C8	Sputum	Day 2	Imipenem/cilastatin → tigecycline	After specimen collection (same hospital day)	Day 2 / 9 days

Empirical carbapenem plus colistin was started after collection of the first positive specimen. Once carbapenem resistance was reported, the carbapenem was discontinued and replaced, according to the susceptibility report, by ampicillin-sulbactam or tigecycline; colistin was continued throughout. Treatment duration followed the institutional protocol (7–10 days). Days are counted from admission (hospital day 1). AST, antimicrobial susceptibility testing.

Supplementary Table S2. Comparison of patients with and without complete detailed data (detailed-data subset vs. remainder of the full cohort), including CRAB status.

Variable	Detailed-data subset (*n* = 95)	Without complete detailed data (*n* = 300)	Missing (subset / without complete data)	Comparison / interpretation
Source-verified records	95/95 (100%)	300/300 (100%)	0/95 / 0/300	Core variables verified against medical charts
Reason for group assignment	All article-specific variables available	Non-core article-specific variables not extracted	0/95 / 0/300	—
Age, years	72 (64–83)	70 (61–81)	0/95 / 0/300	$p = 0.182$ (Mann–Whitney U)
Male sex	68 (71.6%)	201 (67.0%)	0/95 / 0/300	$p = 0.450$ (Fisher exact)
Admission SpO_2, %	92 (91–95)	93 (90–96)	0/95 / 0/300	$p = 0.215$ (Mann–Whitney U)
ICU admission	9 (9.5%)	26 (8.7%)	0/95 / 0/300	$p = 0.836$ (Fisher exact)
In-hospital death	15 (15.8%)	39 (13.0%)	0/95 / 0/300	$p = 0.496$ (Fisher exact); OR 1.25 (exact 95% CI 0.61–2.47)
CRAB isolation	8 (8.4%)	25 (8.3%)	0/95 / 0/300	$p = 1.000$ (Fisher exact)
Blood culture performed	95 (100%)	300 (100%)	0/95 / 0/300	Complete testing logs available
Respiratory culture performed	28 (29.5%)	78 (26.0%)	0/95 / 0/300	$p = 0.509$ (Fisher exact)
Cardiovascular disease	53 (55.8%)	158 (52.7%)	0/95 / 0/300	$p = 0.638$ (Fisher exact)
Diabetes mellitus	48 (50.5%)	142 (47.3%)	0/95 / 0/300	$p = 0.638$ (Fisher exact)
Chronic kidney disease	17 (17.9%)	48 (16.0%)	0/95 / 0/300	$p = 0.638$ (Fisher exact)

Supplementary Table S3. Microbiological sampling denominators in the detailed-data subset (*n* = 95)

Specimen	Patients sampled /95	Samples / collection period	Patients with CRAB / sampled	Negative / no specimen / unknown
Blood culture	95 / 95 (100%)	12 July – 25 September 2022	1 / 95 (1.1%)	70 negative, 24 other pathogens / 0 missing
Sputum	16 / 95 (16.8%)	12 July – 25 September 2022	3 / 16 (18.8%)	13 negative/other pathogens

Specimen	Patients sampled /95	Samples / collection period	Patients with CRAB / sampled	Negative / no specimen / unknown
Tracheal aspirate	12 / 95 (12.6%)	12 July – 25 September 2022	4 / 12 (33.3%)	8 negative/other pathogens
Any respiratory specimen	28 / 95 (29.5%)	12 July – 25 September 2022	7 / 28 (25.0%)	21 negative/other pathogens

Supplementary Table S4. Laboratory susceptibility methodology and phenotype

Measure / Agent	Method / EUCAST version	Number tested	Results / Phenotype	Unavailability / Clinical notes
Species ID	MALDI-TOF MS	8 / 8	8/8 *Acinetobacter baumannii*	Confirmed
Meropenem / Imipenem	Broth microdilution / EUCAST v12.0	8 / 8	8/8 Resistant (100%)	MIC values not archived; no confirmatory method documented
Colistin	Broth microdilution (EUCAST)	8 / 8	8/8 Susceptible (100%, MIC ≤ 2 mg/L)	Continued throughout treatment (10 days; 9 days in C8)
Ampicillin-Sulbactam	Disk diffusion; no EUCAST v12.0 *Acinetobacter* breakpoint	8 / 8	Inhibition zones 16–21 mm; susceptible by CLSI M100 criteria applied by the local laboratory (no EUCAST v12.0 breakpoint)	Replaced the carbapenem after AST in 6/8; combined with colistin
Tigecycline	Broth microdilution; no EUCAST v12.0 *Acinetobacter* breakpoint	8 / 8	MIC 1.0–2.0 mg/L (broth microdilution); susceptible by FDA criteria (≤ 2 mg/L) applied by the local laboratory (no EUCAST or CLSI *Acinetobacter* breakpoint)	Replaced the carbapenem after AST in 2/8 (C6, C8); combined with colistin
Cefiderocol	N/A	0 / 8	Not evaluated / Not administered (0%)	Agent unavailable/not used in cohort

Molecular testing	PCR / Sequencing	0 / 8	Not documented	Carbapenemase genes (OXA/NDM) not tested

Supplementary Table S5. Respiratory infection versus colonization assessment (retrospective chart-based adjudication according to IDSA/ATS definitions; C5, bloodstream isolate, not included)

Case	Imaging (X-ray/CT)	Compatible symptoms	Respiratory deterioration	Culture quantity / decision	Adjudication
C1	No new focal infiltrate	Mild cough, afebrile	Stable SpO_2 (93% room air)	Moderate growth; target colistin+sulbactam	Colonization
C2	New bilateral lower-lobe consolidation	Purulent endotracheal secretions, fever 38.8°C	PaO_2/FiO_2 drop < 200 (mechanical ventilation)	Heavy growth (>10^6 CFU/mL); targeted regimen	VAP (Pneumonia)
C3	Baseline chronic changes, no new opacity	Scant sputum, no fever	Stable SpO_2 on 2L oxygen	Low growth; targeted colistin+sulbactam	Colonization
C4	New right-basal alveolar infiltrate	Productive cough, fever 38.5°C	Increased O_2 requirement (from 2L to 8L/min)	Moderate growth; targeted colistin+sulbactam	HAP (Pneumonia)
C6	New left-middle zone patchy opacity	Fever 38.2°C, dyspnea	Tachypnea, SpO_2 86% on room air	Heavy growth; targeted colistin+tigecycline	HAP (Pneumonia)
C7	No new infiltrates	Minimal secretions	Stable SpO_2 on low-flow oxygen	Low growth; targeted colistin+sulbactam	Colonization
C8	Diffuse interstitial COVID-19 changes only	Mucoid sputum	Stable O_2 saturation	Low growth; targeted colistin+tigecycline	Colonization

Supplementary Table S6. Primary (full-cohort) and secondary or sensitivity analyses of the association between CRAB isolation and outcomes.

Analysis	Eligible denominator / timing	Estimate (95% CI)	p and BH q	Interpretation / Model notes
Full-cohort crude mortality (primary)	*N* = 395 full cohort (33 exposed, 362 unexposed)	OR = 4.35 (exact conditional 95% CI: 1.81–10.02)	*p = 0.0004888654; q = 0.0039*	Primary analysis (adopted during peer review).
Full-cohort Firth adjusted mortality model	*N* = 395 (adjusted for age and admission SpO_2)	Adjusted OR = 3.82 (95% CI: 1.62–9.02)	*p = 0.0021; q = 0.0084*	Penalized likelihood model accounting for baseline severity.
Detailed-data subset crude mortality	*N* = 95 detailed-data subset (8 exposed, 87 unexposed)	OR = 6.91 (Exact conditional 95% CI: 1.08–41.79)	*p = 0.0199; q = 0.0318*	Secondary analysis (primary analysis of the original submission).
Selection audit	*N* = 395 (95 with vs 300 without complete detailed data)	OR = 1.25 (exact 95% CI: 0.61–2.47)	*p* = 0.496; not part of BH family	Mortality in patients with vs without complete detailed data.
MICE sensitivity model	*N* = 395 full cohort (m = 20 imputations)	Pooled OR = 5.82 (95% CI: 0.94–36.02)	*p = 0.0584; q = 0.0779*	Imputes missing article-specific clinical, laboratory and treatment covariates across the full cohort under MAR; CRAB status known for all 395 patients (not imputed).
Detailed-data subset Firth model	*N* = 95 (adjusted for age and admission SpO_2)	Adjusted OR = 5.12 (95% CI: 0.88–29.84)	*p = 0.0688; q = 0.0786*	Penalized likelihood model accounting for baseline severity.
Day-6 landmark mortality	*N* = 88 alive and hospitalized at day 6 (4/8 CRAB vs 8/80 non-CRAB; exposure = detection on or before day 6)	HR = 5.67 (95% CI: 1.63–19.72)	*p = 0.0063; q = 0.0168*	Conditional landmark analysis excluding early mortality.

Analysis	Eligible denominator / timing	Estimate (95% CI)	p and BH q	Interpretation / Model notes
Respiratory-sampled subset	*N* = 28 patients with respiratory cultures	OR = 3.20 (95% CI: 0.52–19.60)	*p = 0.2081; q = 0.2081*	Exploratory subset restricted to sampled respiratory cases.
Total length of stay	*N* = 8 CRAB cases vs 87 controls	Hodges–Lehmann location shift: +11.5 days (95% CI: 3.2–19.8)	*p = 0.0124; q = 0.0248*	Total length of stay (Mann–Whitney U); temporal direction not established.
Exploratory contrasts (Tables 1–4)	Table 1: 9 tests; Table 2: 15 tests; Table 3: 15 tests; Table 4: 7 tests	See Tables 1–4	BH applied within each table; q-values in table footnotes and Table 4	*q < 0.05 only for carbapenem, vancomycin and anakinra (Table 3); full-cohort baseline contrasts (Table 1): q < 0.05 for SpO_2, respiratory sampling, ICU and mortality; no subset baseline or secondary mortality contrast had q < 0.05.*

OR, odds ratio; HR, hazard ratio; CI, confidence interval; MICE, multiple imputation by chained equations; SpO_2, pulse oximetric oxygen saturation; BH, Benjamini–Hochberg; FDR, false discovery rate. q-values are Benjamini–Hochberg-adjusted across the eight primary and sensitivity estimates (selection audit excluded). The full-cohort primary analysis was adopted during peer review; the detailed-data subset crude analysis was the primary analysis of the original submission, and the penalized, landmark, imputation and multiplicity analyses were added post hoc (main text, Section 2.7).

Supplementary Table S7. Variable-specific summary of missing data in the full cohort (*N* = 395).

Variable	Detailed-data subset (*n* = 95)	Without complete detailed data (*n* = 300)	Full cohort (*N* = 395)	Handling in analyses
CRAB status	0 (0%)	0 (0%)	0 (0%)	Complete; not imputed
In-hospital death	0 (0%)	0 (0%)	0 (0%)	Complete; primary outcome
Age, sex, admission SpO_2	0 (0%)	0 (0%)	0 (0%)	Complete; adjustment covariates
ICU admission; blood and respiratory culture sampling	0 (0%)	0 (0%)	0 (0%)	Complete

Cardiovascular disease, diabetes, chronic kidney disease	0 (0%)	0 (0%)	0 (0%)	Complete
Residence, body-mass index, smoking	0 (0%)	300 (100%)	300 (75.9%)	Imputed (MICE) in the sensitivity analysis
COVID-19 vaccination history	0 (0%)	300 (100%)	300 (75.9%)	Imputed (MICE) in the sensitivity analysis
Supplemental oxygen and its duration	0 (0%)	300 (100%)	300 (75.9%)	Imputed (MICE) in the sensitivity analysis
Remdesivir, anakinra, systemic corticosteroids	0 (0%)	300 (100%)	300 (75.9%)	Imputed (MICE) in the sensitivity analysis
Antibacterial courses and timing; length of hospitalization	0 (0%)	300 (100%)	300 (75.9%)	Imputed (MICE) in the sensitivity analysis
Admission laboratory values (leukocytes, CRP, procalcitonin, ferritin)	0 (0%)	300 (100%)	300 (75.9%)	Imputed (MICE) in the sensitivity analysis
Hospital day of first CRAB isolation (CRAB cases only)	0/8 (0%)	25/25 (100%)	25/33 (75.8%)	Required for the landmark analysis; not imputed

Values are number (%) of patients with missing data. Core variables were source-verified against medical charts for all 395 patients (Table S2). Detailed article-specific variables were extracted and verified only for the 95 patients of the detailed-data subset; they were not extracted for the remaining 300 patients. CRP, C-reactive protein; ICU, intensive care unit; MICE, multiple imputation by chained equations; SpO_2, pulse oximetric oxygen saturation.
